# A Novel Nomogram for Predicting Post-Operative Sepsis for Patients With Solitary, Unilateral and Proximal Ureteral Stones After Treatment Using Percutaneous Nephrolithotomy or Flexible Ureteroscopy

**DOI:** 10.3389/fsurg.2022.814293

**Published:** 2022-04-15

**Authors:** Jian-Xuan Sun, Jin-Zhou Xu, Chen-Qian Liu, Yang Xun, Jun-lin Lu, Meng-Yao Xu, Ye An, Jia Hu, Cong Li, Qi-Dong Xia, Shao-Gang Wang

**Affiliations:** Department and Institute of Urology, Tongji Medical College, Tongji Hospital, Huazhong University of Science and Technology, Wuhan, China

**Keywords:** sepsis, percutaneous nephrolithotomy, flexible ureteroscopy, nomogram, urolithiasis, albumin

## Abstract

**Background:**

The postoperative sepsis is a latent fatal complication for both flexible ureteroscopy (fURS) and percutaneous nephrolithotomy (PNL). An effective predictive model constructed by readily available clinical markers is urgently needed to reduce postoperative adverse events caused by infection. This study aims to determine the pre-operative predictors of sepsis in patients with unilateral, solitary, and proximal ureteral stones after fURS and PNL.

**Methods:**

We retrospectively enrolled 910 patients with solitary proximal ureteral stone with stone size 10–20 mm who underwent fURS or PNL from Tongji Hospital's database, including 412 fURS cases and 498 PNL cases. We used the least absolute shrinkage and selection operator (LASSO) regression and multivariate logistic regression analysis to identify the risk factors for sepsis. Finally, a nomogram was assembled utilizing these risk factors.

**Results:**

In this study, 49 patients (5.4%) developed sepsis after fURS or PNL surgery. Lasso regression showed postoperative sepsis was associated with gender (female), pre-operative fever, serum albumin (<35 g/L), positive urine culture, serum WBC (≥10,000 cells/ml), serum neutrophil, positive urine nitrite and operation type (fURS). The multivariate logistic analysis indicated that positive urine culture (odds ratio [*OR*] = 5.9092, 95% *CI* [2.6425–13.2140], *p* < 0.0001) and fURS (*OR* = 1.9348, 95% *CI* [1.0219–3.6631], *p* = 0.0427) were independent risk factors of sepsis and albumin ≥ 35g/L (*OR* = 0.4321, 95% *CI* [0.2054–0.9089], *p* = 0.0270) was independent protective factor of sepsis. A nomogram was constructed and exhibited favorable discrimination (area under receiver operating characteristic curve was 0.78), calibration [Hosmer–Lemeshow (HL) test *p* = 0.904], and net benefits displayed by decision curve analysis (DCA).

**Conclusions:**

Patients who underwent fURS compared to PNL or have certain pre-operative characteristics, such as albumin <35 g/L and positive urine culture, are more likely to develop postoperative sepsis. Cautious preoperative evaluation and appropriate operation type are crucial to reducing serious infectious events after surgery, especially for patients with solitary, unilateral, and proximal ureteral stones sized 10–20 mm.

## Introduction

Urolithiasis is one of the most common urologic diseases and its prevalence rate varies from 1 to 20% depending on the geographical, climatic, ethnic, dietary, and genetic factors (1). Evidence have shown that both the incidence and prevalence rates of urolithiasis are rising during the last three decades worldwide, such as North America, Europe, Australia, and Asia (2). Moreover, the recurrence rate of urolithiasis after initial formation is reported to approach 50% at 5 years and 80–90% at 10 years (3), thus bringing a significant economic burden to both individuals and government, with an additional $1.24 billion/year estimated by 2030 (4). The treatment options for urolithiasis include medicine and surgery, such as extracorporeal shock wave lithotripsy (ESWL), ureteroscopy (URS), and percutaneous nephrolithotomy (PNL), among which both PNL and flexible ureteroscopy (fURS) are standard procedures to remove 10–20 mm ureteral stone (5).

Sepsis is a complicated disorder characterized by the dysregulated host response to an infection, which can finally lead to acute organ dysfunction and even death (6). It is also one of the most common life-threatening complications for both PNL and fURS, with an estimated incidence rate of 0.3–7.6% and 3–5%, respectively (7, 8). It seems that different operation types may influence the incidence rate of sepsis. Except for operation type, other variables have been reported to be independent risk factors for sepsis after PNL or fURS, such as staghorn calculi, positive midstream urine culture, stone size, and albumin–globulin ratio (AGR) (7, 9, 10). However, previous studies have focused more on the risk factors of sepsis for patients undergoing a specific type of operation, but few of them paid attention to the risk factors of post-operation sepsis for patients with stone solitary, unilateral, and proximal ureteral sized 10–20 mm, for whom both PNL and fURS are appropriate. Thus, identifying risk factors that are possible to lead to fatal sepsis after operation for these patients is necessary. Therefore, in this study, we evaluated the latent risk factors of sepsis for patients with solitary, unilateral, and proximal ureteral stones sized 10–20 mm after treatment using PNL or fURS, and constructed a novel nomogram to predict the probability of sepsis for these populations.

## Materials and Methods

The research was approved by the Ethics Committee of Tongji Medical College (2019S1035). We retrospectively enrolled 910 patients with solitary proximal ureteral stone with stone size between 10 and 20 mm from January 2012 to December 2018, including 412 fURS cases and 498 PNL cases. The inclusion criteria were (1) unilateral, solitary, and proximal ureteral stones; (2) PNL or fURS was performed to treat urolithiasis; (3) stone size was between 10 mm and 20 mm; (4) age ≥18 years. The exclusion criteria were anatomical abnormality: solitary kidney, horseshoe kidney, transplant kidney, and kidney duplication. Patients' imaging, including abdominal computed tomography (CT), confirmed the presence and size of the proximal ureteral stone (above the fourth lumbar spine or in the ureteropelvic junction). Types of anesthesia, pre-operative antibiotics, and operation details were identified and recorded. The post-operative data of patients were collected, revised, and recorded.

The primary outcome was postoperative sepsis. Postoperative sepsis was defined as the concurrence of infection and at least two of the following criteria within 48 h of surgery according to the 2001 International Sepsis Definitions Conference,: (1) heart rate > 90/min, (2) respiratory rate > 20/min, (3) body temperature >38°C, and (4) leukocyte count <4,000 cells/μl or > 12,000 cells/μl (11).

The patients' information was retrospectively collected from our hospital's database. The pre-operative factors were recorded, such as age, gender, body mass index (BMI), pre-operative comorbidities (diabetes, coronary heart disease, and hypertension), stone size and laterality, presence of hydronephrosis and indwelling stent, hematological tests (serum white blood cell [WBC], neutrophil, and lymphocyte), serum biochemical tests (creatinine, cholesterol, albumin, and globulin), urine tests (urine WBC and urine nitrite), urine culture, and American Society of Anesthesiologists (ASA) score. All the patients used a 14-Fr ureteral access sheath (Cook Medical, Bloomington, IN) during the fURS in our hospital to decrease the intrarenal pressure. For PNL, we used ultrasound for pre-procedural imaging evaluations and used 20-Fr (micro) sheath for most patients, and 18- or 22-Fr sheath for a small percentage of patients. And we used a standard sized ureteroscope (Storz) for PNL for its small diameter, so intrarenal pressure could be better controlled. All the surgeries were performed by experienced urologists. Operation time was defined as the period between the commencement of operation and the end of anesthesia. The laboratory tests were routinely performed and obtained for all patients. Patients with infectious indicators, such as fever, leukocytosis, and positive urine culture, received at least a full antibiotic regimen for 7 days until the tests turned negative. Otherwise, single-dose administration was applied for antibiotic prophylaxis. A double-J stent was used routinely after surgery in our hospital.

All statistical analyses were performed using Statistical Product and Service Solutions (SPSS) version 25.0 and R software 4.1.1. The cut-off value was determined by the Youden Index. Student's *t-*test was used to detect differences between continuous variables (expressed by mean ± standard deviation [*SD*]) with a normal distribution. Continuous variables with a skewed distribution were shown as median (interquartile range [IQR]) and compared by the Mann–Whitney *U*-test. The chi-square test or Fisher's exact test was used to detect the difference between groups with categorical variables (expressed by proportions). The Kruskal–Wallis H rank sum test was utilized to compare the ranked ordinal variables (expressed by proportions). The least absolute shrinkage and selection operator (LASSO) regression method was applied to preliminarily screened out the candidates of risk factors for further multivariate analysis. Candidates with non-zero coefficients are selected to establish the LASSO model (12). The multivariable logistic regression method was used to identify the independent risk factors of sepsis. The predictive nomogram was constructed based on the proportionally converting each regression coefficient in multivariate logistic regression to a 0–100-point scale (13). Then we used the calibration curve, the receiver operating characteristic (ROC) curve, and decision curve analysis (DCA) to evaluate the predictive performance of this model (14). A calibration curve was generated with 1,000 bootstrap samples to reduce the overfit bias and the Hosmer–Lemeshow (HL) test was applied to assess calibration. An insignificant result of this test indicated good calibration. We plotted the ROC curve and calculated the area under the curve (AUC) of this model and the other three models established based on univariate to assess the discrimination. Ultimately, we used DCA to estimate the net benefits at different threshold probabilities and assessed the clinical usefulness of this nomogram. The difference was considered statistically significant when *p*-value is <0.05.

## Results

**Figure 1:**
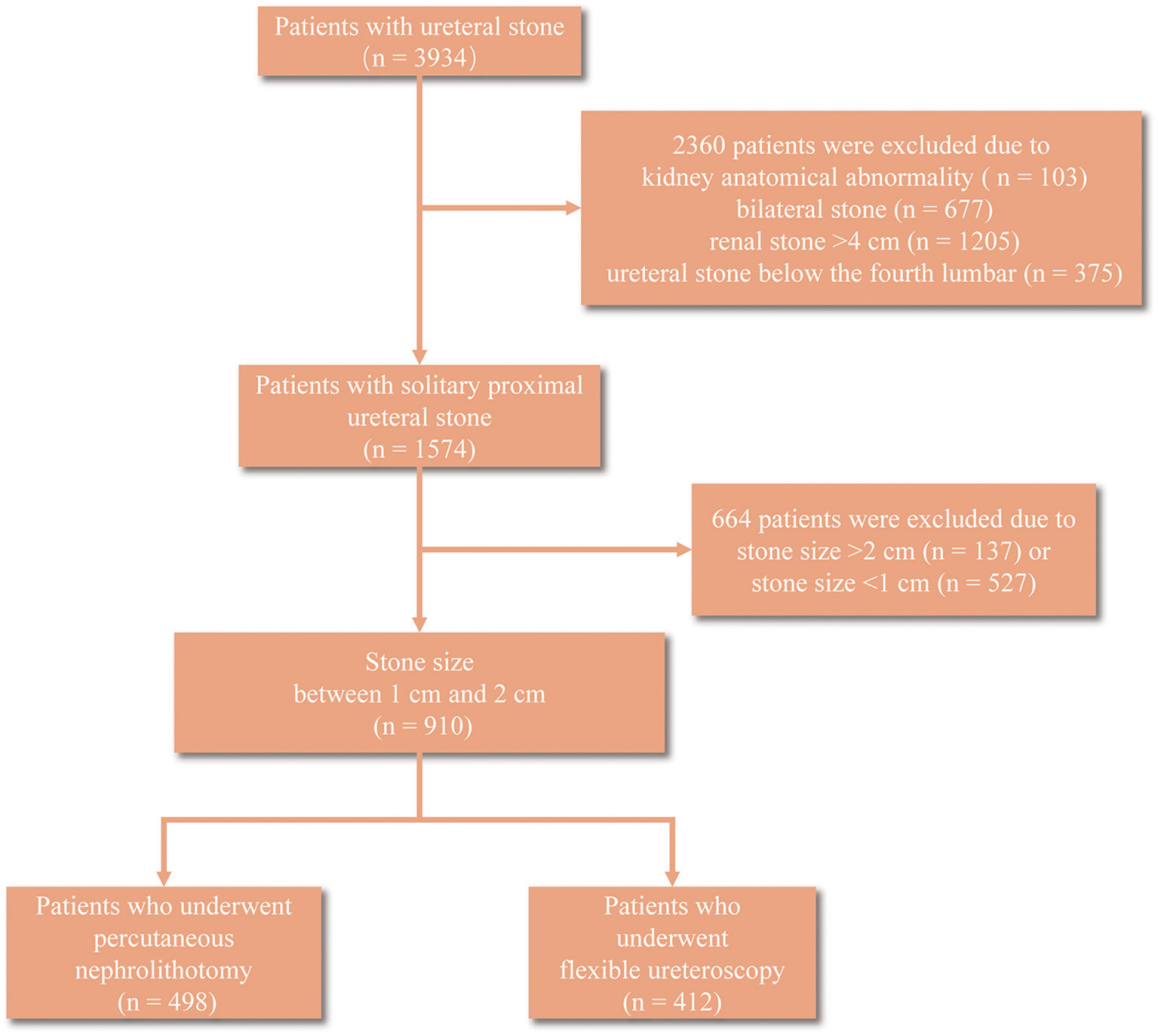
The screening flowchart.

A total of 3,934 patients with ureteral stone were collected, among which 2,360 were excluded after screening due to kidney anatomical abnormality (*n* = 103), bilateral stone (*n* = 677), renal stone > 4 cm (*n* = 1205), and ureteral stone below the fourth lumbar (*n* = 375) (Figure 1). Ultimately, we identified 910 patients with 10–20 mm solitary proximal ureteral stone for the next analyses, and 49 patients developed sepsis among them. The basic characteristics of these patients were summarized in Table 1. Patients who developed sepsis were characterized by female, lower albumin (<35 g/L), higher globulin (≥30 g/L), lower AGR (<1.5), higher rate of pre-operative fever, higher serum WBC (≥10,000 cells/ml), higher serum neutrophil, higher rate of positive urine culture, higher urine WBC, and a higher rate of positive urine nitrite. Notably, the proportion of patients who underwent PNL was lower in the sepsis group (44.9%) compared to the non-sepsis group (55.3%) but the difference had no statistical significance (*p* = 0.155). No significant difference was also observed in age, BMI, operation time, stone size, stone laterality, pre-operative comorbidities, such as hypertension, coronary disease and diabetes, serum cholesterol and creatinine, and ASA score (*p* = 0.050).

**Table 1:** Basic characteristics of including patients.

**Variable**	**All patients (*n* = 910)**	**Sepsis (*n* = 49)**	**Non-sepsis (*n* = 861)**	***P* value**
Age, median (IQR) (years)	51.00 (43.00, 59.00)	53.00 (46.00, 60.00)	50.00 (42.00, 59.00)	0.070
BMI, median (IQR) (kg/m^2^)	23.94 (21.88, 25.85)	23.61 (21.60, 26.30)	23.94 (21.88, 25.85)	0.909
Operation time, median (IQR) (min)	85.00 (66.25, 110.00)	90.00 (70.00, 113.00)	85.00 (66.00, 110.00)	0.134
Gender, *n* (%)				0.001
Male	588 (64.6)	21 (42.9)	567 (65.9)	
Female	322 (35.4)	28 (57.1)	294 (34.1)	
Stone size, median (IQR) (mm)	12.15 (10.00, 15.00)	12.00 (11.00, 15.00)	12.30 (10.00, 15.00)	0.776
Stone laterality, *n* (%)				0.286
Left	476 (52.3)	22 (44.9)	454 (52.7)	
Right	434 (47.7)	27 (55.1)	407 (47.3)	
Indwelling stent, *n* (%)				0.522
Yes	63 (6.9)	5 (20.2)	58 (6.7)	
No	847 (93.1)	44 (89.8)	803 (93.3)	
Hydronephrosis, *n* (%)				0.090
Yes	131 (14.4)	3 (6.1)	128 (14.9)	
No	779 (85.6)	46 (93.9)	733 (85.1)	
Hypertension, *n* (%)				0.809
Yes	210 (23.1)	12 (24.5)	198 (23.0)	
No	700 (76.9)	37 (75.5)	663 (77.0)	
Coronary heart disease, *n* (%)				0.567^a^
Yes	15 (1.6)	1 (2.0)	14 (1.6)	
No	895 (98.4)	48 (98.0)	847 (98.4)	
Diabetes, *n* (%)				
Yes	74 (8.1)	4 (8.2)	70 (8.1)	1.000
No	836 (91.9)	45 (91.8)	791 (91.9)	
Serum cholesterol, *n* (%)				0.746
<5.17 mmol/L	804 (88.4)	44 (89.8)	760 (88.3)	
≥5.17 mmol/L	106 (11.6)	5 (10.2)	101 (11.7)	
Serum creatinine, *n* (%)				0.521
Normal	772 (84.8)	40 (81.6)	732 (85.0)	
Abnormal	138 (15.2)	9 (18.4)	129 (15.0)	
Albumin, *n* (%)				<0.001
<35 g/L	460 (50.5)	38 (77.6)	422 (49.0)	
≥35 g/L	450 (49.5)	11 (22.4)	439 (51.0)	
Globulin, *n* (%)				0.039
<30 g/L	572 (62.9)	24 (49.0)	548 (63.6)	
≥30 g/L	338 (37.1)	25 (51.0)	313 (36.4)	
AGR, *n* (%)				0.001
<1.5	605 (66.5)	43 (87.8)	562 (65.3)	
≥1.5	305 (33.5)	6 (12.2)	299 (34.7)	
Pre-operative fever, *n* (%)				<0.001
Yes	56 (6.2)	10 (20.4)	46 (5.3)	
No	854 (93.8)	39 (79.6)	815 (94.7)	
WBC, *n* (%)				<0.001
<10,000 cells/mL	838 (92.1)	38 (77.6)	800 (92.9)	
≥10,000 cells/mL	72 (7.9)	11 (22.4)	61 (7.1)	
Neutrophil, median (IQR) (10^9^ cells/L)	3.57 (2.71, 4.80)	4.51 (2.79, 6.78)	3.54 (2.71, 4.72)	0.014
Lymphocyte, median (IQR) (10^9^ cells/L)	1.84 (1.43, 2.26)	1.83 (1.32, 2.20)	1.84 (1.45, 2.26)	0.225
Urine culture, n (%)				<0.001
Positive	100 (11.0)	23 (46.9)	77 (8.9)	
Negative	810 (89.0)	26 (53.1)	784 (91.1)	
Urine WBC, *n* (%)				<0.001^b^
-	428 (47.0)	13 (26.5)	415 (48.2)	
+	219 (24.1)	6 (12.2)	213 (24.7)	
++	111 (12.2)	10 (20.4)	101 (11.7)	
+++	152 (16.7)	20 (40.8)	132 (15.3)	
Urine WBC, median (IQR) (cells/hpf)	51.95 (19.72, 163.05)	201.00 (44.90, 643.50)	49.50 (18.90, 139.20)	<0.001
Urine nitrite, *n* (%)				<0.001
Positive	55 (6.0)	15 (30.6)	40 (4.6)	
Negative	855 (94.0)	34 (69.4)	821 (95.4)	
ASA score, *n* (%)				0.050^b^
1	378 (41.5)	14 (28.6)	364 (42.3)	
2	510 (56.0)	33 (67.3)	477 (55.4)	
3	21 (2.3)	2 (4.1)	19 (2.2)	
4	1 (0.1)	0 (0.0)	1 (0.1)	
operation type, *n* (%)				0.155
PNL	498 (54.7)	22 (44.9)	476 (55.3)	
fURS	412 (45.3)	27 (55.1)	385 (44.7)	

*IQR, interquartile range; BMI, body mass index; AGR, albumin globulin ratio; WBC, white blood cell.*

a *Tested with Fisher's Exact Test.*

b *Tested with Kruskal-Wallis H Test*.

**Figure 2:**
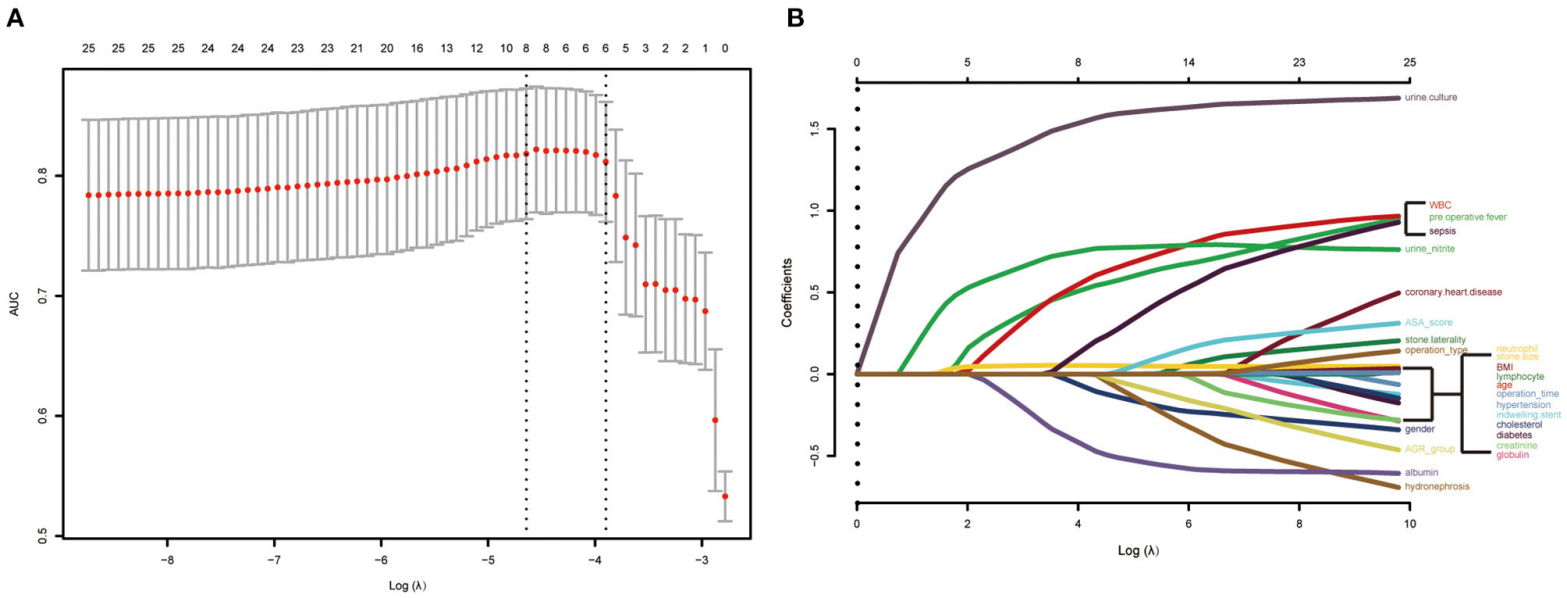
Risk predictors selection using the least absolute shrinkage and selection operator (LASSO) logistic regression model. **(A)** Optimal predictor (lambda) selection in the LASSO model with fivefold cross-validation by minimum criteria. The area under the receiver operation characteristic curve was plotted vs. log (lambda). Dotted vertical lines were drawn at the optimal values by using the minimum criteria and the 1 SE of the minimum criteria; **(B)** LASSO coefficient profiles of the 25 predictors. A coefficient profile plot was developed according to the log (lambda) sequence. A vertical line was drawn at the value selected with fivefold cross-validation, where optimal lambda resulted in 8 predictors with nonzero coefficients (lambda = 0.009629).

In the lasso regression, we screened out eight variables, such as gender, pre-operative fever, serum albumin, urine culture, serum WBC, serum neutrophil, urine nitrite, and operation type (Figures 2A,B) (Table S1). The multivariate logistic analysis indicated that two variables were independent risk factors of sepsis (Table 2): positive urine culture (*OR* = 5.9092, 95% *CI* [2.6425–13.2140], *p* < 0.0001) and fURS (*OR* = 1.9348, 95% *CI* [1.0219–3.6631], *p* = 0.0427), and albumin ≥ 35g/L (odds ratio [*OR*] = 0.4321, 95% *CI* [0.2054–0.9089], *p* = 0.0270) was independent protective factor of sepsis. Gender (*p* = 0.2702), pre-operative fever (*p* = 0.1055), serum WBC (*p* = 0.0987), serum neutrophil (*p* = 0.2814), and urine nitrite (*p* = 0.0508) were not considered as independent risk factors.

**Table 2:** Multivariable logistic regression analysis of predictors of sepsis.

**Variables**	**B**	**SE**	**OR**	**95% CI**	***P* value**
Female	0.3656	0.3316	1.4414	[0.7525–2.7611]	0.2702
Pre-operative fever	0.7356	0.4545	2.0867	[0.8563–5.0854]	0.1055
Albumin≥35g/L	−0.8392	0.3794	0.4321	[0.2054–0.9089]	0.0270
WBC ≥10,000 cells/mL	0.9313	0.5640	2.5378	[0.8403–7.6649]	0.0987
Neutrophil	0.0501	0.0465	1.1102	[0.9179–1.3428]	0.2814
Positive urine culture	1.7765	0.4106	5.9092	[2.6425–13.2140]	<0.0001
Positive urine nitrite	0.8940	0.4578	2.4449	[0.9967–5.9969]	0.0508
fURS	0.6600	0.3257	1.9348	[1.0219–3.6631]	0.0427

*WBC, white blood cell; B, regression coefficient; SE, standard error; OR, Odds Risk; CI, confidence interval*.

**Figure 3:**
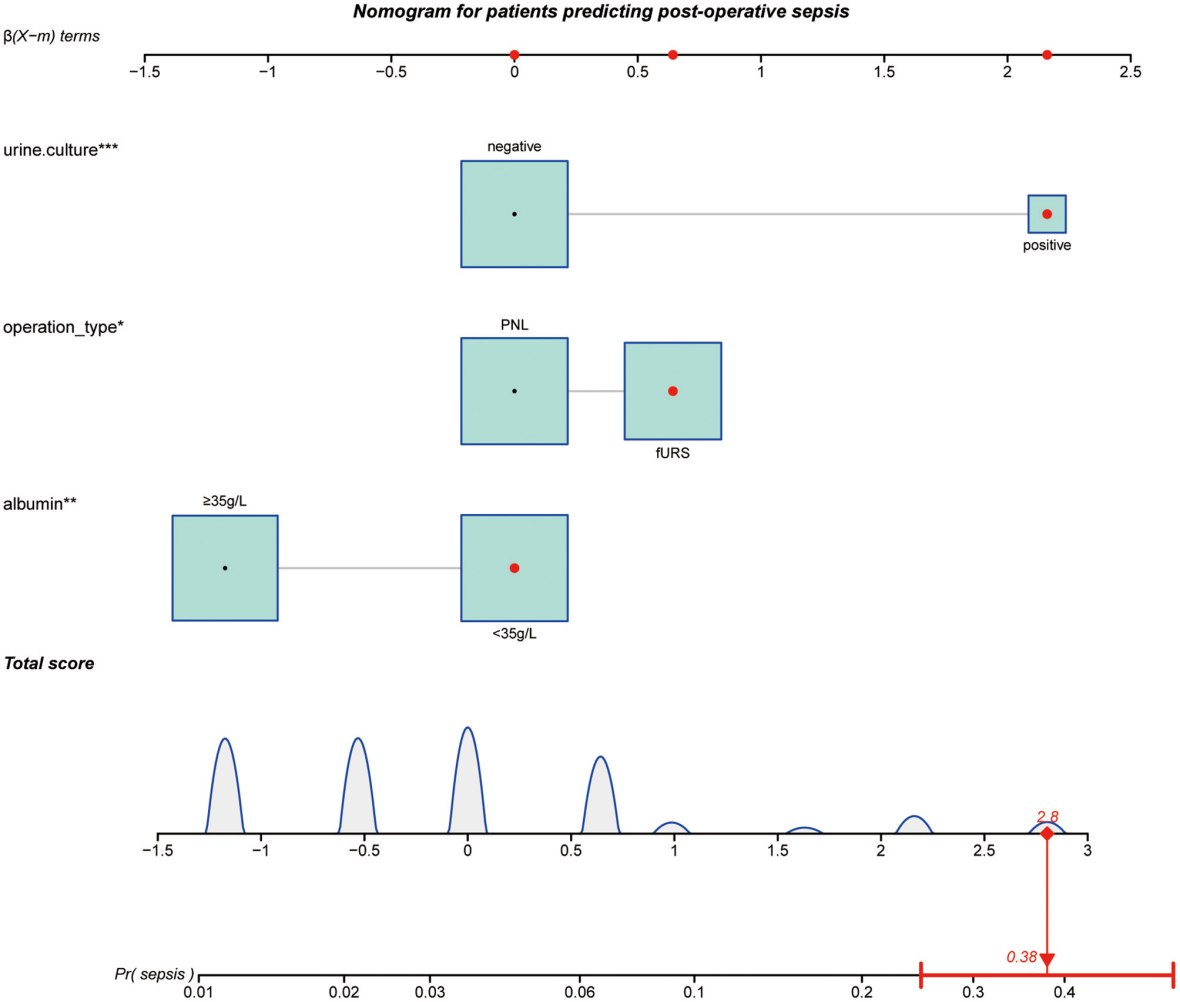
Nomogram for patients predicting postoperative sepsis. Urine culture, operation type, and albumin are marked as “points.” Total points by adding the four points can predict sepsis rate. One patient whose urine culture was positive, operation type was fURS and serum albumin level <35 g/L was randomly selected for analysis. After adding the score of each item, a total score of 2.8 and the corresponding sepsis rate of 0.38 (95% *CI* [0.25–0.53]) were obtained. The asterisks represented the statistical p value (*P < 0.05; **P < 0.01; ***P < 0.001).

**Figure 4:**
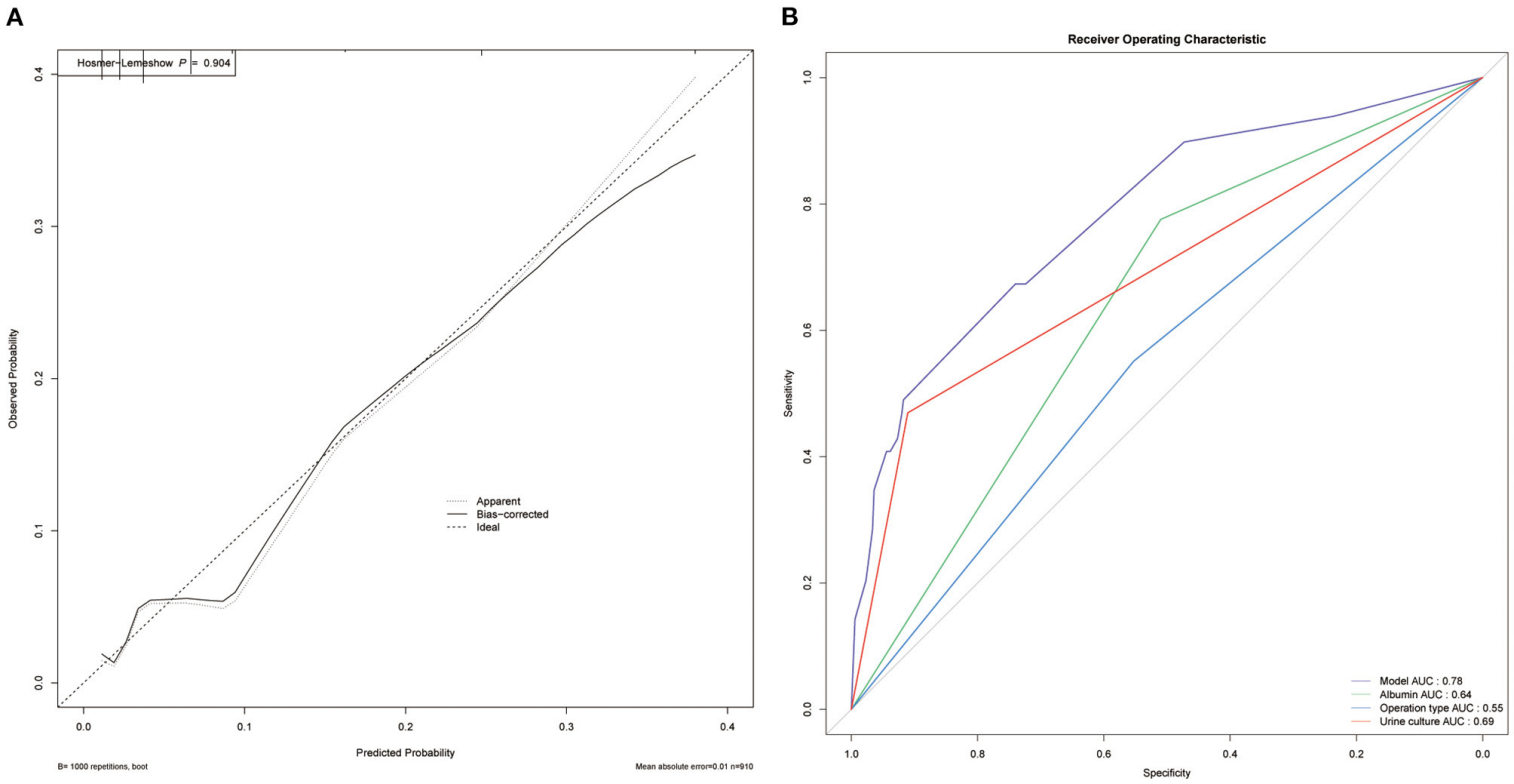
Evaluation of the predictive performance. **(A)** Calibration curve. The The HL test with insignificant *p*-value indicates good fitting of the model. **(B)** Receiver operating characteristic (ROC) curve. The area under curve (AUC) for the model is 0.78, which showed a favorable ability of discrimination.

**Figure 5:**
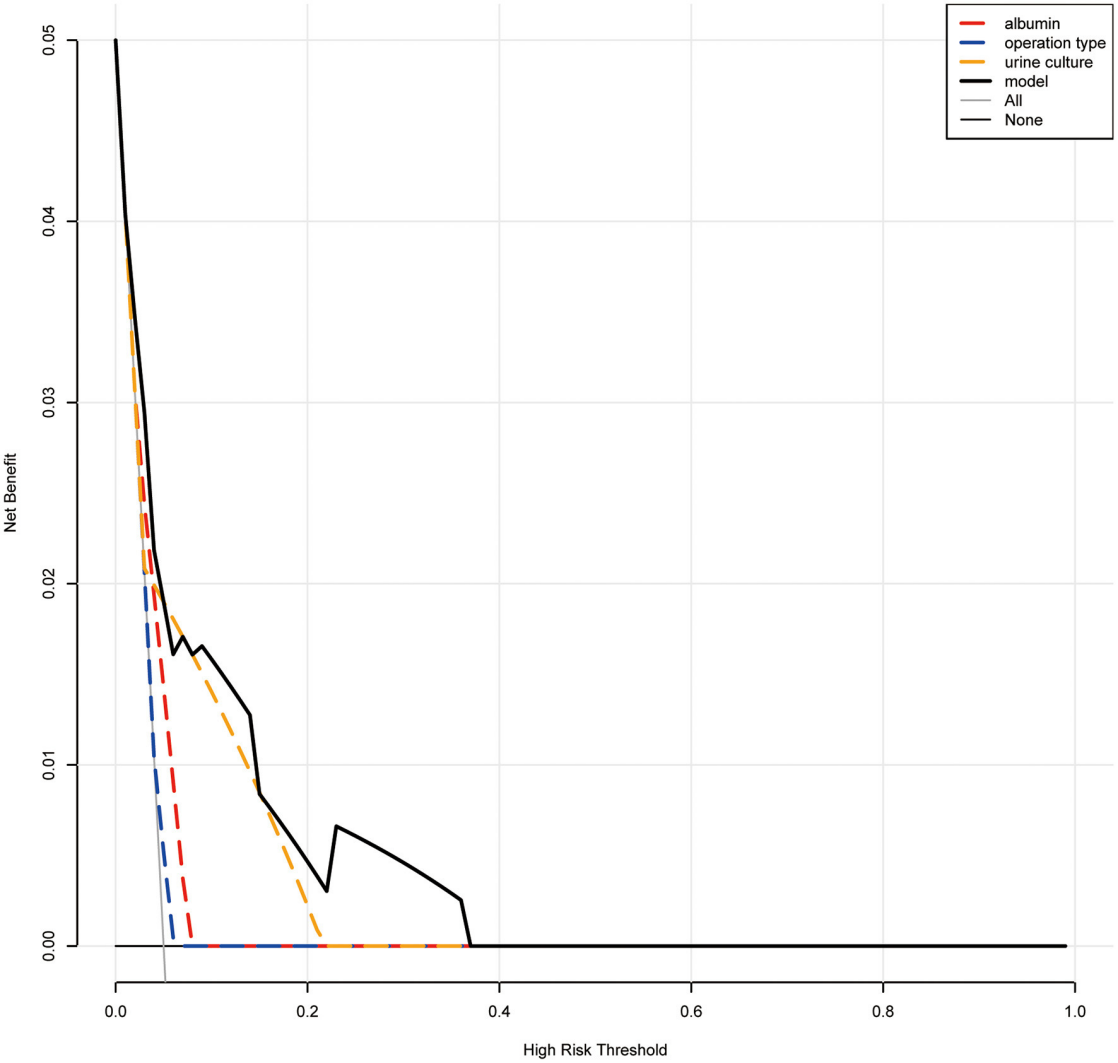
Decision curve analysis (DCA). When the risk threshold is around 10–38%, the net benefit of application of the model on taking measures is greater than the “treat-all-patient” or “treat-none” scheme. In addition, utilization of albumin, urine culture, or operation type alone is inferior to the model.

Based on the multivariate analysis, we established a nomogram prediction model to calculate the cumulative probability of post-operative sepsis (Figure 3). The total points were calculated by adding the respective points obtained from the three factors. The corresponding sepsis rate is indicated by the total points axis. We randomly chose one patient who had positive urine culture and serum albumin level <35 g/l, and underwent fURS. After adding the score of each item, we obtained a total score of 2.8 and the corresponding sepsis rate of 0.38 (95% *CI* [0.25–0.53]). The calibration curve showed good fitting of the model with no statistical significance (*p* = 0.904) through the HL test (Figure 4A). The AUC of the ROC curve for our model was 0.78, which was larger than the AUC of other models established by univariate, such as serum albumin (*AUC* = 0.64), operation type (*AUC* = 0.55), and urine culture (*AUC* = 0.69), and indicated better discrimination of our model (Figure 4B). The DCA of the model showed a threshold probability of 10–38%, in which our model was able to identify patients with solitary proximal ureteral stone between 10 and 20 mm who might develop post-operative sepsis superior to the “treat-all-patients” or “treat-none” schemes and other models based on univariate (Figure 5).

## Discussion

Sepsis, a syndrome of physiologic, pathologic, and biochemical abnormalities induced by infection (15), is one of the most severe postoperative complications for both PNL and fURS (7, 16). A systematic review from the EAU Section of Urolithiasis (EULIS) has reported a total incidence rate of 0.51% (*n* = 126) in the cohort, including patients from 14 articles, accounting for 6.5% of all the complications reported (17). In our study, the incidence rate of sepsis was 5.4%, which was higher than the data reported. This may result from the fact that our hospital treated more critically ill patients with worse overall conditions. Previous studies have focused on a group of patients with a certain type of surgery or stone and identified several valuable independent risk factors. Gao et al. have found that preoperative Cr level and positive pre-operative multidrug-resistant (MDR) urine culture were independent risk factors in patients with struvite stones following PNL (18). Jiang et al. have identified positive urine culture, serum calcium, and urine pH that were significantly connected with the incidence of sepsis after PNL (19). Moreover, Ozgor and his colleagues demonstrated that patients with ≤ 40 years old, operation time ≥60 min, and presence of renal abnormality were remarkably associated with infectious events following fURS (16). Besides, other factors, such as hydronephrosis, diabetes, C-reactive protein, and so on, which may influence the systematic condition and local urological condition, have been reported to result in infectious events after surgery (20, 21).

In this article, based on the LASSO regression and multivariable logistic regression, we identified that albumin level <35 g/l and fURS and positive urine culture were independent risk factors of post-operative sepsis for patients with solitary, unilateral, and proximal ureteral stone sized 10–20 mm. Then a nomogram was generated utilizing these risk factors. The calibration curves indicated a good consistency between nomogram-predicted sepsis probability and the actual outcome. The ROC curve exhibited higher AUC than other models constructed based on univariate, which showed a favorable ability of discrimination of our model. And the results of the DCA curve showed patients would benefit more from clinical decisions made using this model than treat none, treat all patients or other models when we set the threshold probability between 10 and 38% to predict the probability of sepsis (22, 23). Since the incidence of sepsis is far <10%, our model has a significant value in clinical practice.

Albumin is the critical component in the serum to preserve the colloid osmotic pressure. It also plays an important role in the transport of water-insoluble substances in plasma, signal transduction, buffering acid-base properties, and anti-inflammatory and anti-oxidative effects (24). The permeability barrier of vessel consists of not only endothelial cells but also glycocalyx, which is a layer of a few microns on endothelial cells and composed of glycosaminoglycans (25). Owing to both having negative charges, albumin tends to accumulate at the outer layer of glycocalyx, thus forming a second barrier to prevent fluid leakage from the vessel. However, in sepsis, the vascular permeability is increased since the glycocalyx and endothelium are damaged or dysfunctional, but evidence showed that the albumin might protect against the loss of glycocalyx and reduce its damage in shock models (26), therefore, decreasing the fluid leakage. Moreover, albumin can also maintain the integrity and function of the proximal tubule cells to exhibit the renal protection function in sepsis (27), and it is reported that hypoalbuminemia was a risk factor for renal failure (28). Previous studies have found high high-Sensitive C-Reactive protein/albumin ratio and low AGR could both serve as an independent risk factor of sepsis after PNL and fURS respectively (9, 29). Therefore, it is reasonable that hypoalbuminemia is also a latent predictor for post-operative sepsis in our study. Some studies found that the supplement of albumin to patients with sepsis showed a significant advantage, while others showed that albumin was not associated with the reduction of all-cause mortality for adults with sepsis of any severity (with or without baseline hypoalbuminemia) (30, 31). So, whether it is necessary to supply albumin to patients during post-operative sepsis is still controversial.

Our previous studies have demonstrated that PNL was superior to fURS in reducing the incidence of sepsis in treating patients with solitary proximal ureteral stone and positive urine culture (32). In general, PNL showed a better stone-free rate but higher complication rate compared to fURS and had more advantages in treating large stone sized more than 20 mm (33, 34), but the operation time and hospital stay were controversial in different studies and might depend on the stone size and the proficiency of operators (34–36). Therefore, it was usually difficult for surgeons to make a choice between PNL and fURS, especially for patients with solitary, unilateral, and proximal ureteral stones sized 10–20 mm, for which PNL and fURS were both suitable (5). Here, our work found PNL might be a better surgery strategy to reduce severe postoperative infectious complications, especially for those who have positive urine culture and low levels of albumin before surgery.

Unlike albumin and fURS, positive urine culture has been proved to serve as an independent risk factor of sepsis in many previous studies (7, 10, 18). It is recommended by the EAU guideline for urolithiasis that urinary tract infection should always be treated before stone removal (5). However, for those with negative urine culture, the use of antibiotics for prophylaxis was also beneficial for reducing the rate of post-operative fever and other complications after PNL (37). And a single dose administration was demonstrated to be sufficient (38).

There are still some limitations in this study, mainly resulting from its retrospective nature, which may bring in selection bias. Moreover, our samples are from the database of a single institution, thus lacking external validation. The sample size in our study is relatively large to make our results more stable. However, the number of specific patient population in our samples is relatively small, such as patients with diabetes, which was reported to serve as a predictor for infectious complications in previous studies (21). Therefore, further prospective and multicenter studies, including patients with more comprehensive characteristics, are needed. Although a novel definition of sepsis (Sepsis-3) has been put forward using the Sequential [Sepsis-related] Organ Failure Assessment (SOFA) score, the definition we used (Sepsis-2) showed an advantage in early identification and had higher sensitivity. But the Sepsis-3 definition could select patients with more severe cases of sepsis (39). Besides, the latest study had reported that the performance of SOFA was only slightly greater than qSOFA and SIRS in predicting the septic shock after PCNL (40). Therefore, we would further collect more clinical samples and construct a predictive model using the Sepsis-3 definition to compare with the model based on Sepsis-2 in the future.

## Conclusion

A novel nomogram is constructed to predict sepsis in patients with solitary, unilateral, and proximal ureteral stones sized 10–20 mm after treatment using PNL or fURS. Patients who underwent fURS compared to PNL or have certain pre-operative characteristics, such as albumin <35 g/L and positive urine culture, are more likely to develop post-operative sepsis.

## Data Availability Statement

The original contributions presented in the study are included in the article/Supplementary Material, further inquiries can be directed to the corresponding authors.

## Ethics Statement

The studies involving human participants were reviewed and approved by Ethics Committee of Tongji Medical College, Huazhong University of Science and Technology (2019S1035). The patients/participants provided their written informed consent to participate in this study. Written informed consent was obtained from the individual(s) for the publication of any potentially identifiable images or data included in this article.

## Author Contributions

J-XS and Q-DX analyzed the data, wrote the manuscript, and drew the figures. S-GW, CL, and JH designed the study. C-QL and J-ZX collected and assembled the data. YX, J-lL, M-YX, and YA contributed to the critical revision of the manuscript. All authors contributed to the article and approved the submitted version.

## Funding

This work was supported by the Natural Science Foundation of China (81772729), Key Research and Development Program of Hubei Province (General Project) (2020BCB052), and the Innovative Entrepreneurship Training Program of HUST (S202110487434).

## Conflict of Interest

The authors declare that the research was conducted in the absence of any commercial or financial relationships that could be construed as a potential conflict of interest.

## Publisher's Note

All claims expressed in this article are solely those of the authors and do not necessarily represent those of their affiliated organizations, or those of the publisher, the editors and the reviewers. Any product that may be evaluated in this article, or claim that may be made by its manufacturer, is not guaranteed or endorsed by the publisher.
